# Identification of Gut Bacteria such as Lactobacillus johnsonii that Disseminate to Systemic Tissues of Wild Type and MyD88–/– Mice

**DOI:** 10.1080/19490976.2021.2007743

**Published:** 2022-01-13

**Authors:** Sreeram Udayan, Panagiota Stamou, Fiona Crispie, Ana Hickey, Alexandria N. Floyd, Chyi-Song Hsieh, Paul D. Cotter, Orla O’Sullivan, Silvia Melgar, Paul W. O’Toole, Rodney D. Newberry, Valerio Rossini, Ken Nally

**Affiliations:** a APC Microbiome Ireland, University College Cork, Cork, Ireland; b School of Biochemistry and Cell Biology, University College Cork, Cork, Ireland; c Department of Internal Medicine, Division of Gastroenterology, Washington University School of Medicine in St. Louis, St. Louis, MO, USA; d Teagasc Food Research Center, Moorepark, Cork, Ireland; e Department of Internal Medicine, Division of Rheumatology, Washington University School of Medicine, St. Louis, MO, USA; f School of Microbiology, University College Cork, Cork, Ireland

**Keywords:** Gut microbiota, commensal bacteria, symbionts, systemic dissemination, translocation, immunomodulation, *Lactobacillus johnsonii*, MyD88

## Abstract

In healthy hosts the gut microbiota is restricted to gut tissues by several barriers some of which require MyD88-dependent innate immune sensor pathways. Nevertheless, some gut taxa have been reported to disseminate to systemic tissues. However, the extent to which this normally occurs during homeostasis in healthy organisms is still unknown. In this study, we recovered viable gut bacteria from systemic tissues of healthy wild type (WT) and MyD88^−/−^ mice. Shotgun metagenomic-sequencing revealed a marked increase in the relative abundance of *L. johnsonii* in intestinal tissues of MyD88^−/−^ mice compared to WT mice. *Lactobacillus johnsonii* was detected most frequently from multiple systemic tissues and at higher levels in MyD88^−/−^ mice compared to WT mice. Viable *L. johnsonii* strains were recovered from different cell types sorted from intestinal and systemic tissues of WT and MyD88^−/−^ mice. *L. johnsonii* could persist in dendritic cells and may represent murine immunomodulatory endosymbionts.

## Introduction

The current paradigm regarding sampling of luminal material in the gut by mononuclear phagocytes is that migratory intestinal dendritic cells (DCs) can acquire bacteria directly^1^ or indirectly from epithelial cells or macrophages^2,3^ and then migrate in a CCR7-dependent manner^4^ only as far as the gut draining lymph nodes for initiation of appropriate adaptive immune responses.^5,6^ This has led to the current consensus that bacterial members of the gut microbiota are separated from systemic compartments by multiple molecular, cellular, and tissue barriers or ‘firewalls’.^7,8^ However, the interaction of members of the gut microbiota with the host is not restricted to the gastrointestinal tract (GIT) and can also occur after translocation of selected gut bacterial strains to systemic tissues.^9^ There is emerging evidence of the existence of extraintestinal tissue microbiotas in human disease states^10,11^ and human gut symbionts were reported to disseminate from the gut in the absence of an inflammatory response in germ free (GF) mice.^12^ Such interactions can have multiple physiological and pathophysiological effects on the host. For example, *Achromobacter spp., Bordetella spp*. and *Ochrobactrum spp*. can translocate from the gut to lymphoid tissues or the spleen to influence adaptive immunity and prevent dissemination of other luminal bacteria.^13^ Translocation of gut bacteria might also contribute to the vertical transfer of bacterial species from the mother to the neonate prenatally by crossing the maternal placental barrier^14,15^ or postnatally through maternal breast milk transfer.^16,17^

However, it is not known if systemic dissemination of gut bacteria occurs in the absence of perturbation to the host or to the gut barrier. Host MyD88-dependent pattern recognition receptor (PRR) pathways are essential for the containment of gut bacteria in the gut lumen and their exclusion from the systemic compartment.^18^ Studies in MyD88^−/−^ mice have shown increased dissemination of gut resident bacteria to systemic tissues.^19,20^ However, it has not been investigated if bacteria also translocate in WT mice and the specific bacteria that translocate in these mice have not been identified. The main objective of this study was to systematically investigate this dissemination phenomenon and its underpinning mechanisms in mice during the basal homeostatic state. Using a validated aseptic culture-based approach we recovered gut-associated bacteria from systemic tissues and from sorted tissue cells of WT and MyD88^−/−^ mice. *Lactobacillus johnsonii* was identified as the most abundant bacterial species in systemic tissues of mice with higher abundance observed in Myd88^−/−^ mice in comparison to WT mice. *L. johnsonii* was able to persist intracellularly in DCs and induce cytokine responses in these cells. We also found that *L. johnsonii* was able to migrate to systemic tissues in monocolonized germ-free WT mice and studies in specific-pathogen-free (SPF) CRR7 knockout (KO) mice indicated that this is CCR7 independent.

## Results

### Viable gut-associated bacteria such as *Lactobacillus johnsonii* are present in systemic tissues of WT and MyD88^−/−^ mice

**Figure 1.**
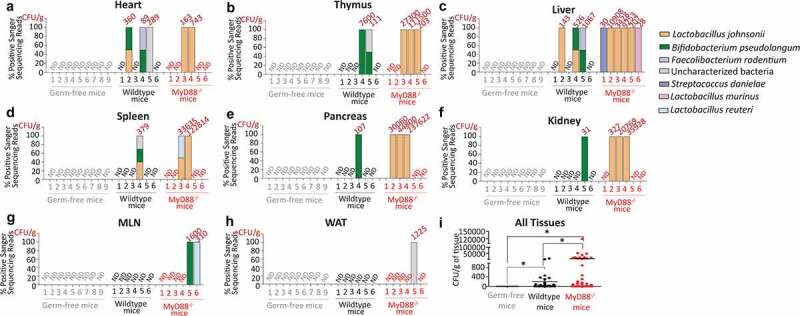
Gut-resident bacteria translocate to systemic tissues of WT and MyD88^−/−^ mice. CFU/g indicates number of colony forming units (CFU) of bacteria recovered per gram of tissue and % Positive sanger sequence reads indicates the presence of different systemic bacteria (sequenced based on morphological differences) in (a) heart, (b) thymus, (c) liver, (d) spleen, (e) pancreas, (f) kidney, (g) mesenteric lymph nodes (MLNs), and (h) white adipose tissues (WAT) in Germ-free, WT and MyD88^−/−^ mice as indicated. Data is from nine individual Germ-free mice, six individual WT, and six individual MyD88^−/−^ mice (all males, 21–23 week age). (i) Total bacterial load indicated as CFU/g recovered from different systemic tissues of WT and Myd88^−/−^ mice as indicated. Statistical analyses were performed by Two- tailed Student's *t* test using GraphPad Prism. *p* < .05 (denoted by *) was considered statistically significant. ND denotes “No detectable bacteria”.

To identify gut-associated bacteria residing in systemic tissues of mice, we initially used a workflow involving standard sterile operating procedures, called workflow 1 (Supplementary material 1). However, this was not sufficient to avoid contamination of tissues isolated from germ free mice by microbes derived from the immediate environment (Supplementary material 2 and 3). Therefore, we used a modified workflow – which included an antibacterial wash step for euthanized mice before dissection and a gentamicin wash step for tissues – to ensure the absence of environmental (skin, air, and work surfaces) microbial contaminants. Using this modified workflow – workflow 2 – we isolated only 1 colony of *Bacillus sps*. from a total of 108 plated whole tissue homogenates (Supplemental material 1) from 9 germ free mice (Figure 1a-h). Subsequently, workflow 2 was used for all further experiments.

We recovered several colonies of distinct gut-associated bacterial species from the different systemic tissues of WT and MyD88^−/−^ mice (Figure 1a-h). We also recovered specific bacteria that are classified here as “uncharacterized bacteria”. This is based on previous reports where they were found at increased levels in the intestine of mice with Salmonella-induced enterocolitis^21^ (Figure 1a,d); in the intestine of mice with diet induced obesity^22^ (Figure 1b); and in colonic tissues of WT and NOD2^−/−^ mice with Dextran Sodium Sulfate (DSS)-induced colitis^23^ (Figure 1h). Notably, *L. johnsonii* were recovered from both WT and MyD88^−/−^ mice, but more frequently from systemic tissues of MyD88^−/−^ mice (Figure 1a-f). *Bifidobacterium pseudolongum* and *Lactobacillus reuteri* were recovered from MLNs (Figure 1g) and “uncharacterized bacteria” were recovered from WAT (Figure 1h) of MyD88^−/−^ mice. To assess if bacteria translocated from gut to systemic tissues, germ-free mice were gavaged with *L. johnsonii* isolated from liver of WT mice. This strain colonized intestinal tissues (stomach, small intestine, cecum, colon, and stool) and translocated, albeit at lower extent, to liver, pancreas, kidney, MLN and WAT tissues of germ-free mice monocolonized with *L. johnsonii* (Supplementary material 4). The overall recoverability of bacterial colonies and the extent of systemic dissemination was significantly higher in MyD88^−/−^ mice in comparison to WT mice (Figure 1i).

### Higher systemic dissemination of *Lactobacillus johnsonii* in MyD88^−/−^ mice is a reflection of its higher relative abundance in intestinal tissues

**Figure 2.**
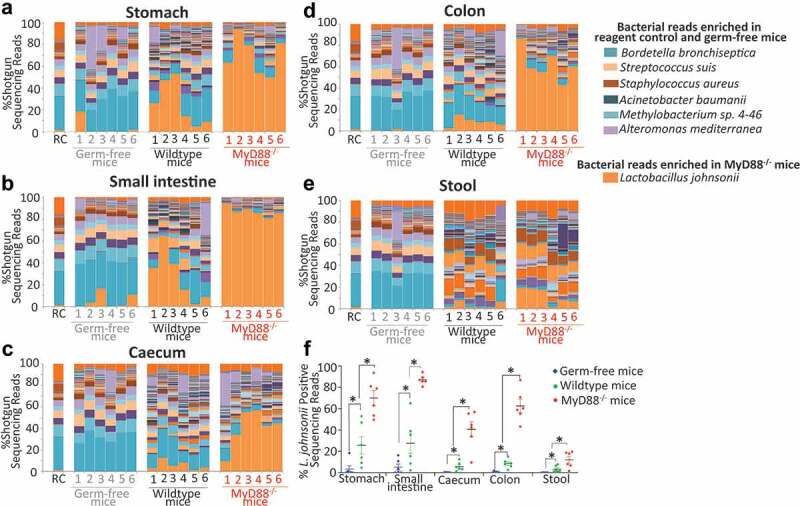
*L. johnsonii* positive sequence reads were higher in MyD88^−/−^ mice intestinal tissues in comparison to WT tissues. Each color in the bar graph represents “% Shotgun sequencing reads” corresponding to different bacterial shotgun reads (denoted by different colors) in (a) stomach, (b) small intestine, (c) cecum, (d) colon, (e) stool for each of the indicated samples (RC – reagent control) of this dataset identified using Kraken. (f) “% Shotgun metagenome read” specifically corresponding to *L. johnsonii* in GF, WT and MyD88^−/−^ mice. A comprehensive list of shotgun metagenome read percentages corresponding to all the bacteria identified from these samples is listed in Supplementary Material 6. Data are from six WT and six MyD88^−/−^ mice (all males, age: 21–23 weeks) and GF mice (n = 6 males, age: 20 weeks). Statistical analyses were performed by two-tailed Student's t test using GraphPad Prism. *p* < .05 (denoted by *) was considered statistically significant.

To investigate if the higher recoverability of *L. johnsonii* from the systemic tissues of MyD88^−/−^ mice might be due to its higher relative abundance in the GIT of these mice in comparison to WT animals, total tissue DNA was isolated from the stomach, small intestine (SI), cecum, colon and stool of both mouse strains and subjected to shotgun metagenomic sequencing (Figure 2a-f). In addition, as controls total DNA was isolated from the gut tissues of GF mice and DNA extraction kits to identify potential environmental/reagent contaminants (Figure 2a-f). The raw sequencing reads from tissue samples were at least 75-fold higher than the kit-reagent control (Supplementary material 5). The average microbial sequence reads in intestinal tissues and stool samples were higher in WT and MyD88^−/−^ mice in comparison to germ-free mice (Supplementary material 5). Extraction reagents used in genomic DNA extraction (referred here as reagent control) have been reported to consist of contaminating microbial reads.^24^ We identified shotgun reads positive for bacteria such as *Bordetella bronchiseptica, Streptococcus suis, Staphylococcus aureus, Methylobacterium sp., Alteromonas mediterranea* in all of the tested samples including reagent control and germ-free tissues suggesting they represent the reagent microbiome (Figure 2a-f). However, WT and MyD88^−/−^ mice tissue samples consisted of more diverse and distinct microbial reads (Figure 2a-f) which were positive for different gut-resident bacteria (Supplementary material 6). The proportion of sequencing reads corresponding to *L. johnsonii* was substantially higher in the GIT tissues (stomach, SI, cecum, and colon) and stool of MyD88^−/−^ mice in comparison to their proportions in WT mice (Figure 2f). In addition, higher systemic dissemination of *L. johnsonii* was observed in MyD88^−/−^ mice compared to WT mice.

### Gut-associated bacteria are located intracellularly and can be cultured from cells sorted from intestinal tissue of WT and MyD88^−/−^ mice

Since all tissues isolated for recovery of viable bacteria had been washed in 20 µg/mL of the cell-impermeable antibiotic gentamicin before homogenization and plating, any bacteria recovered from these tissues were most likely either resistant to gentamicin or located inside host cells. Indeed, none of the culturable bacteria recovered from systemic tissues were able to grow in bacteriological broth media supplemented with gentamicin at concentrations of 20 µg/mL or higher (Supplementary material 7). We reasoned that these bacteria were most likely located intracellularly in the systemic tissues. Therefore, we investigated if gut-associated bacteria could be recovered from different cell types: DCs (CD45^+^CD64^−^CD11C^+^MHCII^+^), macrophages (CD45^+^CD64^+^), epithelial cells (CD45^−^EpCAM^+^), leukocytes (CD45^+^CD64^−^MHCII^−^), remaining cells minus leuokocytes- CD45 negative (CD45^−^) sorted from single cell suspensions of SI, colon, MLNs, spleen and thymus incubated in gentamicin (Supplementary material 8). Several gut-resident bacteria including *L. johnsonii, L. reuteri, L. murinus, Cutibacterium acnes, Bifidobacterium pseudolongum, E. hirae*, and *Faecalibaculum rodentium* were recovered from sorted cells of SI and colon of WT mice. *L. johnsonii* alone was recovered from sorted cells from small intestine and colon of MyD88^−/−^ mice (Table 1). No gut-resident bacteria were recovered from sorted cells of MLNs and spleen in both mouse strains. However, *Cutibacterium acnes* was recovered from epithelial cells, macrophages and leukocytes from thymus of WT mice. *L. johnsonii* was recovered from the percoll fraction of WT thymus that was enriched for leukocytes and T cells. *Stenotrophomonas spp*., a bacterium previously recovered from splenic DCs^25^ (closely related to low virulence pathogen *Stenotrophomonas maltophilia*^26^) was recovered from MLN leukocytes of MyD88^−/−^ mice (Table 1).

**Table 1.** Culturable gut-associated bacteria isolated from FACS sorted cell subsets from the small intestines, colons, and systemic tissues of WT and MyD88^−/−^ mice. Tissues were isolated from six WT and six MyD88^−/−^ mice (all males, age: 23–27 weeks). CFU/1000 indicates colony forming units recovered for every 1000 sorted cells and was calculated by dividing the total number of colonies recovered per cell type with the total no. of cells sorted

Cell type		WT mice			MyD88^−/−^ mice		
		Bacteria recovered	No. of cells sorted	CFU/ 1000 cells	Bacteria recovered	No. of cellssorted	CFU/ 1000 cells
Small intestine	CD45^−^	*L. reuteri,L. murinus,C. acnes*	964,678	0.051	*L. johnsonii*	685,943	0.04
	Epithelial	*L. reuteri,B. pseudolongum*	517996	0.011	*L. johnsonii*	285,739	0.01
	Macrophage	*L. reuteri*	106,358	0.084	-	504	-
	Dendritic cells	*L. reuteri,E. hirae,B. pseudolongum*	105,191	0.076	*L. johnsonii*	110,319	2.9
	Lymphocyte	*L. johnsonii,L. murinus,C. acnes*	500,000	0.02	*L. johnsonii*	249,568	0.02
Colon	CD45^−^	*L. johnsonii,B. pseudolongum,E. hirae,F. rodentium*	514,304	0.04	*L. johnsonii*	218,912	0.027
	Epithelial	*L. murinus,L. reuteri*	500,000	0.026	*L. johnsonii*	3,026	0.013
	Macrophage	*Uncultured bacterium,L. murinus*	41,869	0.047	-	11,772	-
	Dendritic cells	*F. rodentium*	51,566	0.019	*L. johnsonii*	54,426	0.018
	Lymphocyte	*B. pseudolongum*	100,997	0.019	*L. johnsonii*	120,401	0.018
Thymus	CD45^−^	-	819,698	-	-	1,000,000	-
	Epithelial	*C. acnes*	20,659	0.086	-	40,848	-
	Macrophage	*C. acnes*	69,376	0.048	-	27,330	-
	Dendritic cells	-	291,517	-	-	239,551	-
	T cells	*L. johnsonii*, *C. acnes*, *C. granulosum*	-	-	-	-	-
MLN	DC	-	104,543	-	-	134,838	-
	Lymphocyte	-	927,516	-	*Stenotrophomonas sps.*	1,128,704	0.007
	Macrophage	-	16,197	-	-	31,035	-
	CD45^−^	-	175,642	-	-	525,151	-
Spleen	DC	-	660,229	-	-	110,788	-
	Lymphocyte	-	337,012	-	-	44,835	-
	CD45^−^	-	30,000	-	-	34,160	-

### *Lactobacillus johnsonii* isolated from murine systemic tissues can persist and induce cytokine production in innate immune cells

**Figure 3.**
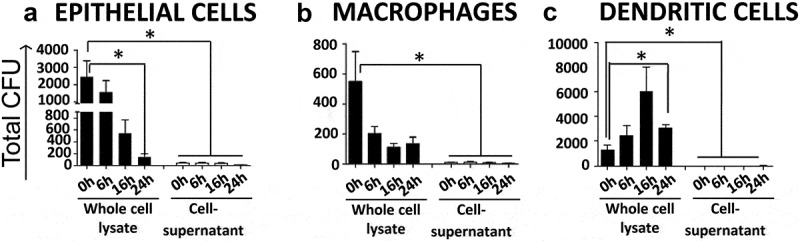
*L. johnsonii* persist in primary DCs but not macrophages or epithelial cells *in vitro. L. johnsonii* was cocultured with (a) CMT93 mouse epithelial cells; (b) BMDMs; and (c) BMDCs at MOI of 10:1, for up to 24 h and cell lysates and cell supernatants were plated separately for the indicated time points. Data are from three independent experiments. Statistical analyses were performed by two-tailed Student's *t* test. *p* < .05 (denoted by *) was considered statistically significant.

*L. johnsonii* was cocultured with the murine intestinal epithelial cell line CMT93, murine bone-marrow derived macrophages (BMDMs) and murine bone-marrow derived dendritic cells (BMDCs) in order to test their ability to persist intracellularly in these cells. The intracellular load of *L. johnsonii* reduced gradually from 0 to 24 h in cocultured epithelial cells (Figure 3a) and macrophages (Figure 3b) but not in DCs (Figure 3c). *L. johnsonii* triggered cytokine (interleukin (IL)-6, IL-10, tumor necrosis factor (TNF)-α, mouse keratinocyte chemoattractant (mKC)) responses in cocultured macrophages (Supplementary material 9A) and DCs (Supplementary material 9B) but they did not trigger mKC (CXCL1) chemokine response in cocultured epithelial cells (Supplementary material 9 C). Since *L. johnsonii* strains were able to persist in cocultured DCs for longer time periods, we investigated if dissemination of *L. johnsonii* from the gut to systemic tissues required migration of DCs from the gut to MLNs using CCR7^−/−^ mice. After cohousing WT and CCR7^−/−^ mice for 12 weeks, to allow potential exchange of the microbiota, no significant difference was observed in the isolation and recovery of bacteria from systemic tissues (Supplementary material 10). *L. johnsonii* was recovered from both groups (Supplementary material 11) indicating that CCR7-dependent migratory DCs were not required for systemic dissemination of *L. johnsonii*.

## Discussion

The main aim of this study was to investigate if gut-associated bacteria are capable of translocating to systemic tissues during homeostasis or are restricted to the gut lumen and associated intestinal tissues. In our current conceptual framework, bacterial members of the gut microbiota are thought to be separated from systemic tissues by multiple molecular, cellular, and tissue barriers.^7,8^ However, this framework is at odds with emerging data which indicates that gut-associated bacteria can be found intracellularly in a wide range of systemic cells and tissues under normal physiological and pathophysiological contexts.^9,27,28^

Previously, meta-analysis of 16S rDNA metagenome sequencing datasets of murine fecal samples had identified *Lactobacillus* as one of most abundant species in the murine gut when compared to human gut.^29^ Specifically, *L. johnsonii* has been reported to inhabit the forestomach, ileum and cecum of mice.^30^ In our study, *L. johnsonii* was the most prominent bacteria recovered from both intestinal and systemic tissues of both WT and MyD88^−/−^ mice. The recovery of *L. johnsonii* was substantially higher in MyD88^−/−^ mice compared to WT mice and this appeared to be a direct reflection of their higher abundance in all intestinal tissues from MyD88^−/−^ mice. *L. johnsonii* was recovered from different sorted cells from intestinal and systemic tissues of WT and MyD88^−/−^ mice suggesting a broad cell tropism. *L. johnsonii* was able to persist for prolonged time periods within BMDCs, suggesting that DCs might be able to retain viable bacteria for longer time periods due to their reported lower phagolysosomal activity compared to macrophages.^31^ Indeed, *Achromobacter spp., Alcaligenes spp., Bordetella spp*. and *Ochrobactrum spp* can persist in DCs^13^ and *E. cloacae*, that can translocate from the gut to MLNs, can be recovered intracellularly from DCs even 60 h after bacterial administration to WT mice.^5^ These observations suggest that DCs might represent an alternative intracellular niche for gut associated bacteria and support the existence of possible molecular mechanisms underpinning the ability of *L. johnsonii* and other bacteria to persist and replicate in DCs.

Many of the bacteria that we recovered from systemic tissues, have been reported to have beneficial host immunomodulatory effects. *L. johnsonii* in particular has been reported to reduce proinflammatory responses in murine liver,^32^ restore normal levels of CD4^+^ and CD8^+^ T cells in spleen,^33^ and trigger differentiation of splenic CD4^+^ T cells into tumoricidal Th17 cells in cyclophosphamide treated tumor mice.^34^ Given that we have also found viable *L. johnsonii* residing in cells and tissues systemically it is tempting to speculate that gut symbionts, such as *L. johnsonii*, could also function as endosymbionts in order to exert their immunomodulatory effects. For the first time we show that, different bacterial members of the murine gut microbiota, predominantly *L. johnsonii*, can translocate from the GIT to systemic tissues in WT and MyD88^−/−^ mice. Our stringent and validated culture-dependent approach will be beneficial to identify the cellular and molecular mechanisms underpinning the gut and systemic immunomodulatory effects of gut symbionts and pathobionts, their systemic dissemination, and their contribution to health and disease.

## Materials and methods

### Mice

Wild type (C57BL/6 J) (cat. number 000664), MyD88^−/−^ (cat. number 009088), and CCR7^−/−^ (cat. number 006621) male mice were purchased from Jackson laboratories. They were housed in UCC Biological Service Units’ animal facility under specific pathogen-free (SPF) conditions, fed a standard pellet diet (Envigo, Cambridgeshire, UK) and tap water ad libitum. The GF mice were bred in-house and maintained in sterile flexible film isolators at UCC Biological Service Units and “Washington University Gnotobiotic Research, Education and Transgenic (GREaT)” animal facility. Germ-free mice were fed SDS RM1 A (P) (Special Diet Services, UK) as their maintenance diet and RM3 A (P) (Special Diet Services UK) as their breeder diet and deionized drinking water ad libitum. All food, water bedding and other supplies needed for GF mice in the isolator were sterilized by autoclaving before being introduced into the isolator. Standard housing and environmental conditions were maintained (temperature 21°C, 12 h light, and 12 h darkness with 50% humidity) in the animal housing facility. All animal experiments were performed in accordance with EU legislation (Directive 2010/63/EU) and the Institutional Animal Care and Use Committee at Washington University School of Medicine for the protection of animals used for scientific purposes. The study was carried out under ethical approval (Euthanasia Only: Application ID 2018/009 and AE19130/PO85) from the Animal Experimentation Ethics Committee of University College Cork.

### Isolation of mouse systemic tissues for the identification of gut-associated bacteria

Mice were euthanized by cervical dislocation prior to dissection and isolation of tissue. For more information on tissue isolation and plating see Supplementary materials (Supplementary Material 1).

### Preparation of single cell suspensions from mouse tissues for fluorescent activated cell sorting (FACS)

Single cell suspension was obtained from SI, colon, MLNs, spleen and thymus. Single cell suspension from SI and colon tissue of euthanized mice were obtained using the lamina propria dissociation kit, mouse (Cat# 130–097-410, Miltenyi) following the manufacturer’s instructions. MLN and spleen tissues were physically homogenized and washed with 1X PBS supplemented with 1% FCS. Red blood cells in spleen were lysed by 10 min incubation at 37°C with 5 mL of 1X Lysebuffer (eBioscience). Furthermore, spleen cells were enriched for dendritic cells (DCs) using mouse pan dendritic cell isolation kit (Miltenyi Biotec). Immune cells were resuspended in 1X PBS supplemented with 1% FCS. Samples were then centrifuged 5 min at 300 × g. Thymi were removed and finely chopped and placed in Roswell Park Memorial Institute medium (RPMI 1640, Sigma) supplemented with 1 mg/mL Collagenase D (Roche), DNase (Sigma), and Dispase (Roche). Pieces were incubated for 30 min at 37°C with gentle shaking. Cells were filtered through 100 µm strainers and cell suspension was gently layered on top of tubes with 52.7% Percoll (Gibco) layered on top of 92.4% Percoll. Tubes were centrifuged at 3000 × g for 30 min at 4°C with brake off. The top fraction of cells was gently collected and resuspended in 1X PBS supplemented with 1% FCS.

### Immunostaining and sorting of single cell suspensions

Monoclonal antibodies were used to stain single cell suspension from different tissues. Prior to FACS analysis isolated cells were incubated with gentamicin (20 µg/ml) for 10 min at 37°C in order to kill potential extracellular bacteria and then washed twice in sterile 1 × PBS. Single cell suspensions were blocked with the monoclonal antibody 2.4 G2 directed against the FcgRIII/II CD16/CD32 (0.5 ng mAb per 106 cells) (Fc block, BD Biosciences) for 15 min followed by immunostaining. 1 × 10^6^ cells were incubated with 0.5 ng of the relevant mAbs (Supplementary material 12) for 20 min at 4°C, and washed again twice.

### Bacterial identification from culture plates by Sanger sequencing

Colony PCR was carried out on the resulting colonies from plated tissue homogenates or from sorted cell populations (lysed in ice cold sterile water) plated in BHI or YCFA, using primers which target full length of bacterial 16S rRNA gene.^35^ PCR products were purified using High Pure PCR product purification kit (Sigma) and identified by full length Sanger-sequencing (Eurofins Genomics).

### Genomic DNA isolation and shotgun metagenomics of murine intestinal tissues and stool

Genomic DNA was extracted from the intestinal tissues by using Qiagen DNeasy Blood & Tissue kit (Qiagen) and extracted from stool samples by using QIAamp Fast DNA Stool mini kit (Qiagen). DNA concentration was estimated using Qubit® (Invitrogen) and metagenomic libraries were prepared using the Nextera XT kit (Illumina) with minor modifications. Briefly, the tagmentation time was increased to 7 min and following addition of indices and purification as described in the manufacturer’s protocol, the average size of each sample was assessed using a High Sensitivity DNA assay on an Agilent bioanalyzer(Agilent) and quantified by Qubit (Invitrogen). The samples were then pooled equimolarly. The concentration of the final pool was determined by qPCR using the Kapa qPCR kit for Illumina (Roche) and sequenced in Teagasc Next Generation Sequencing Facility using a NextSeq™ 500/550 High Output Kit v2 (300 Cycles) kit (Illumina) on the Illumina NextSeq 500 using standard Illumina guidelines. DNA from intestinal tissues of six GF mice and DNA extraction reagents and library preparation (RC – reagent control) were also sequenced as controls.

### Analysis of shotgun metagenomic sequencing data

Taxonomic classifications of Resulting FastQ reads were determined using Kraken 2 and Bracken.^36^ Resulting FastQ reads from sequencing were quality checked by first removing contaminating mouse derived reads using NCBI Best Match Tagger (BMTagger). Resulting reads were trimmed and poor quality and duplicate reads removed using a combination of SAMtools and Picard tools. Taxonomic classifications of trimmed reads were determined using Kraken 2 and Bracken.^36^

### In vitro persistence assay of *L. johnsonii*

*L. johnsonii* strains were cocultured with CMT93 murine intestinal epithelial cell line (ATCC® CCL-223™), BMDMs (bone-marrow-derived macrophages) and BMDCs (bone-marrow-derived DCs). BMDMs and BMDCs were prepared as previously described.^37,38^ *L. johnsonii* strains recovered from systemic tissues were cultured overnight in MRS (DIFCO) under microaerobic conditions (5% CO_2_) at 37°C. Following 2 h of incubation of *L. johnsonii* with the cells, cells were resuspended in 20 µg/mL gentamicin supplemented media for 20 min and then washed with PBS three times and resuspended in fresh cell culture media. At this time point (0 h) cell supernatants were plated in MRS agar. Cells were lysed in sterile ice-cold water and whole cell lysates plated separately as well in MRS agar. Plating of supernatants and whole cell lysates was repeated at 6, 16, and 24 h. Plates were incubated under microaerobic (5% CO_2_) conditions for 48 h before enumerating CFU (colony forming units). All experiments were repeated three times.

### Cytokine secretion of cocultured cells with *L. johnsonii*

Cytokines were measured in the supernatant of CMT93 murine intestinal epithelial cell line, BMDMs or BMDCs cocultured with confluent overnight cultures of *L. johnsonii* strains at an MOI of 10:1 for 24 h. Analysis of mKC was carried out using murine CXCL1 ELISA DuoSet®(R & D systems, Bio-Techne) and analysis of TNF-α, Il-10, Il-12p70, Il-1β, and mKC/CXCL1 cytokines was carried out using 7-plex Uplex MSD assays (MesoScale Discovery, Gaithersburg, MD).

## Supplementary Material

Supplemental Material
