# Supplementary material for: Identification of Gut Bacteria such as Lactobacillus johnsonii that Disseminate to Systemic Tissues of Wild Type and MyD88–/– Mice
Source: Gut Microbes. 2022 Jan 13;14(1):2007743. doi: 10.1080/19490976.2021.2007743 (PMC8765072; doi:10.1080/19490976.2021.2007743)
Supplement: Supplemental Material [file KGMI_A_2007743_SM3086.zip › Supplementary information/Gut Microbes_KGMI_20210378 _Supplementary Material_Final.docx]

**Supplementary Material 1**


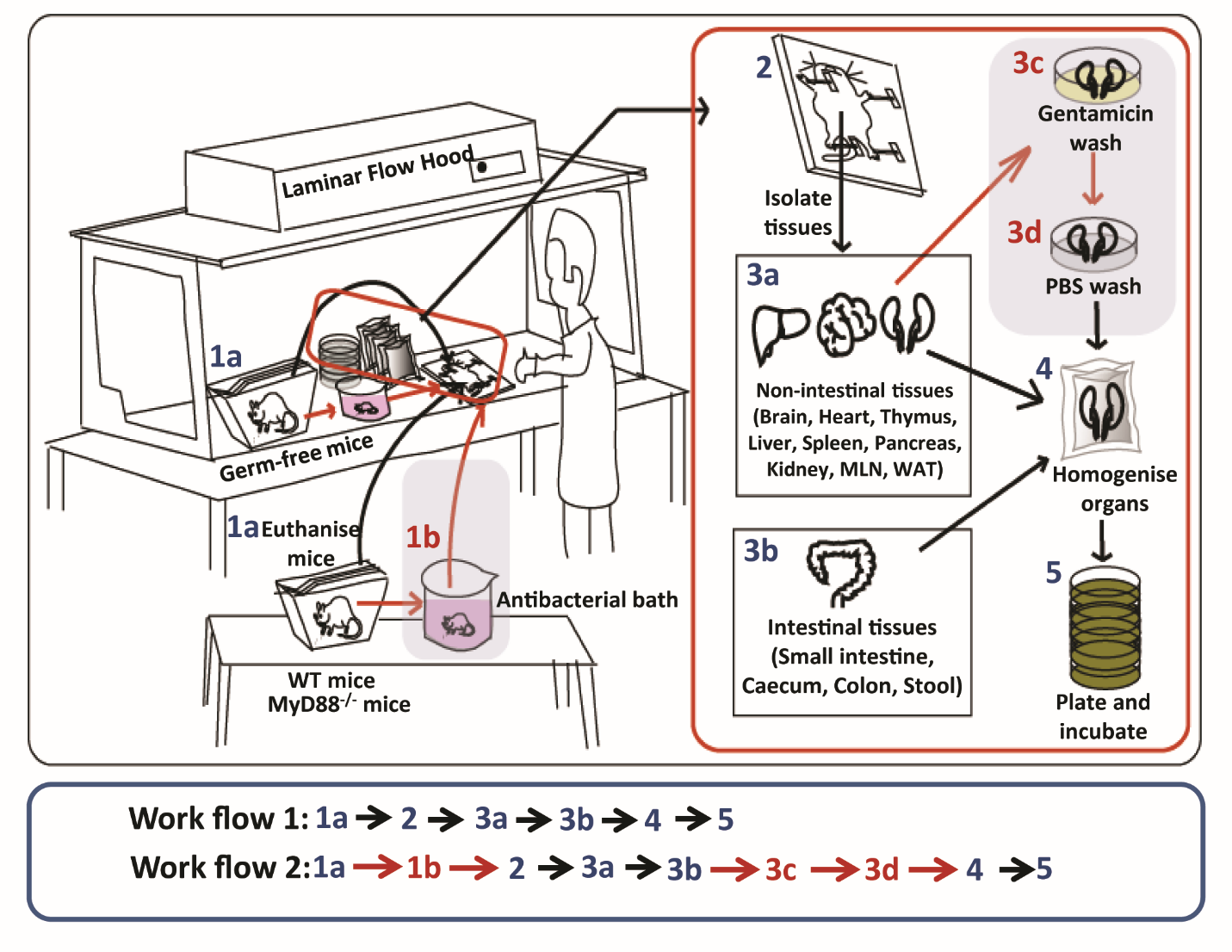


**Supplementary Material 1**. **Workflow 1 and Workflow 2 for isolating bacteria from systemic tissues of mice.** Workflow 1: (1a) Germ free mice are removed from cages placed directly in the laminar hood and directly dissected, whereas WT and MyD88^-/-^ mice are removed from cages outside and then transferred to the laminar flow hood. Mice were euthanized by cervical dislocation. Remainder of the steps (2-5) are carried out in the laminar flow hood (2) Euthanised mice are pinned at fore and hind legs onto a sterile dissection board (3a) Non-intestinal tissues from all mice and ((3b) intestinal tissues from GF mice only are isolated aseptically and transferred to double bagged sterile blender bags filled with 3mL sterile PBS (4) Tissues in the blender bags are homogenised physically by rolling a sterile plastic rod over the blender bag until a uniform tissue homogenate suspension is obtained and (5) 200µL of the homogenate is plated on microbiological nutritional media containing agar coated petri plates and incubated aerobically and anaerobically at 37 °C. Workflow 2: (1a) Same as described in Workflow 1 (1b) Euthanised mice are dipped in 1% antibacterial solution (Virkon®) for 5 minutes (2) Same as described in workflow 1 (3a) Non-intestinal tissues from all mice and ((3b) intestinal tissues from GF mice only are (3c) treated with 10 mL of 20 μg/mL of cell impermeable antibiotic gentamicin for 3 minutes and (3d) briskly washed with 10 mL PBS for 30s and transferred to double bagged sterile blender bags filled with 3mL sterile PBS (4, 5) Same as described in Workflow 1.

**Supplementary Material 2**


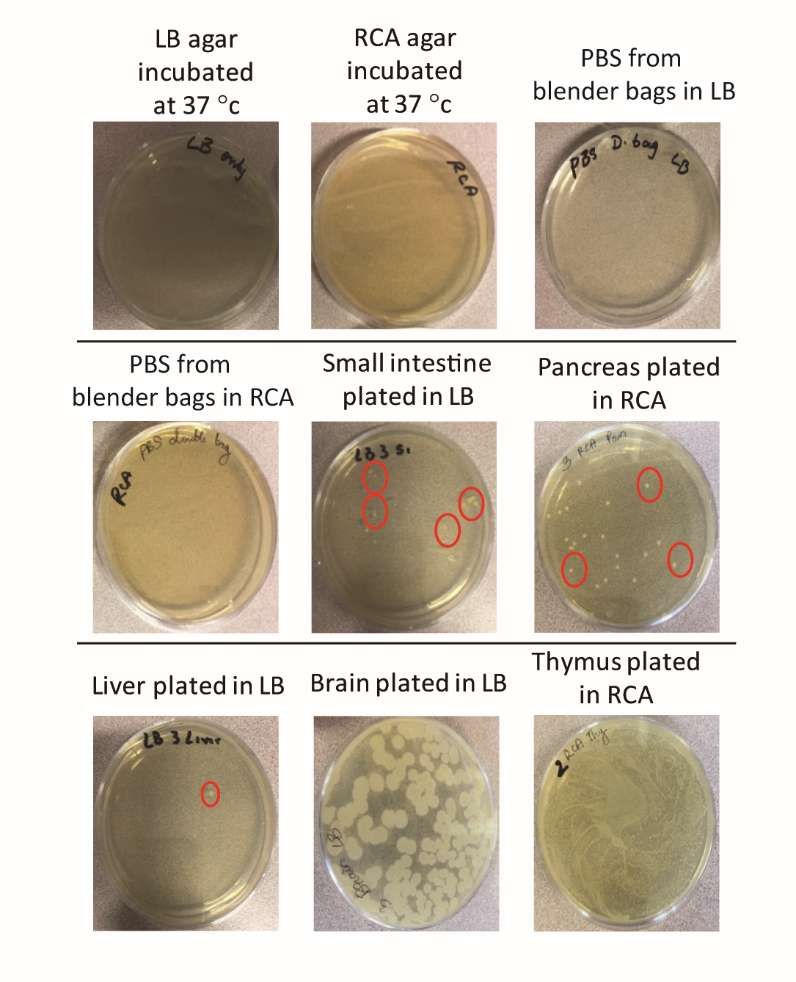


**Supplementary Material 2**: **Standard sterile operating procedures alone are not sufficient to prevent contamination during tissue isolation and processing in GF mice**. Tissues were isolated and plated using Workflow 1 (Supplementary Figure 1). Representative RCA (anaerobically incubated) and LB (aerobically incubated) plates showing lack of colonies in empty agar plates and plates containing PBS (homogenisation buffer) from blender bags, but contaminating colonies are present from small intestine, liver, brain and thymus of GF mice (n=6 males, age: 20 weeks old).

**Supplementary Material 3**

Bacteria identified from systemic tissues of germ-free mice using Workflow 1.

| **Organ** | **Bacteria (Blast hit)** | **% Blast ID** | **Recovered aerobically/anaerobically** | **Possible source(s) of contamination** |
| --- | --- | --- | --- | --- |
| **Liver** | *Bacillus cereus* | 99 | Aerobically and  anaerobically | Environmental |
| **MLN*** | *Bacillus cereus* | 99 | Aerobically and  anaerobically | Environmental |
| **WAT**** | *Micrococcus luteus* | 99 | Aerobically | Environmental/ Mammalian skin microbiota |
| **Kidney** | *Micrococcus luteus* | 99 | Aerobically | Environmental/ Mammalian skin microbiota |
| **Pancreas** | *Staphylococcus capitis* | 99 | Aerobically | Mammalian skin microbiota |
| **Caecum** | *Micrococcus sp.* | 99 | Aerobically | Environmental/ Mammalian skin microbiota |
| **Thymus** | *Staphylococcus capitis* | 99 | Aerobically | Mammalian skin microbiota |
| **Lungs** | *Micrococcus luteus* | 99 | Aerobically | Environmental/ Mammalian skin microbiota |
| **Pancreas** | *Bacillus cereus* | 99 | Aerobically | Environmental |
| **Pancreas** | *Bacillus species* | 99 | Aerobically | Environmental |
| ***MLN- Mesenteric lymph Nodes, **WAT- White adipose tissue** | | | | |

**Supplementary Material 4**

*L. johnsonii* identified in monocolonized germ-free WT mice (n=3), gavaged with 3 x 10^8^ *L. johnsonii*, followed by harvesting, homogenizing and plating of tissues (using Workflow 2), 24 hours later.

| **Organ** | | ***L. johnsonii* positive tissues (n=3)** | **CFU/g** |
| --- | --- | --- | --- |
| Intestinal tissues | Stomach | 3/3 | 213333, 200000, 586666 |
|  | Small Intestine | 3/3 | 228571, 180000, 97142 |
|  | Caecum | 3/3 | 533333, 360000, 400000 |
|  | Colon | 3/3 | 260000, 160000, 170000 |
|  | Stool | 3/3 | 880000, 1440000, 1920000 |
| Systemic tissues | Liver | 1/3 | 0, 143, 0 |
|  | Pancreas | 1/3 | 0, 5500, 0 |
|  | Kidney | 1/3 | 950, 0, 0 |
|  | MLN* | 2/3 | 500, 3100, 0 |
|  | WAT** | 2/3 | 0, 7200, 100 |
| *MLN- Mesenteric lymph Nodes, **WAT- White adipose tissue | | | |

**Supplementary Material 7**


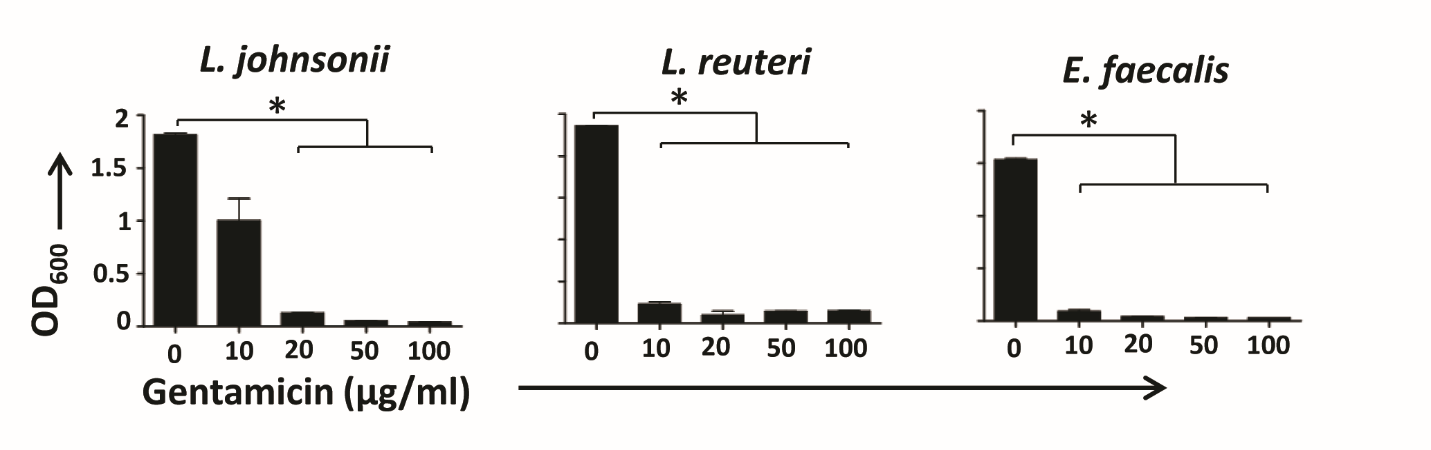


**Supplementary Material 7**: **Bacteria recovered from systemic tissues are sensitive to gentamicin treatment.** Single colonies were inoculated to grow overnight from freshly streaked plates of *L. reuteri*, *L. johnsonii* and *E. faecalis* strains as indicated which were isolated from systemic tissues of WT and MyD88^-/-^ mice. Overnight cultures were subcultured (*Lactobacilli* were cultured in MRS broth and *E. faecalis* in BHI broth) supplemented with varying concentrations of the antibiotic gentamicin at 10 µg/mL, 20 µg/mL, 50 µg/mL, 100 µg/mL and no gentamicin (0µg/mL). OD600 was recorded 6 hours post inoculation of these bacteria. Data are from three independent experiments. Statistical analyses were performed by Two-tailed Students t test using GraphPad Prism. p<0.05 was considered statistically significant.

**Supplementary Material 8**


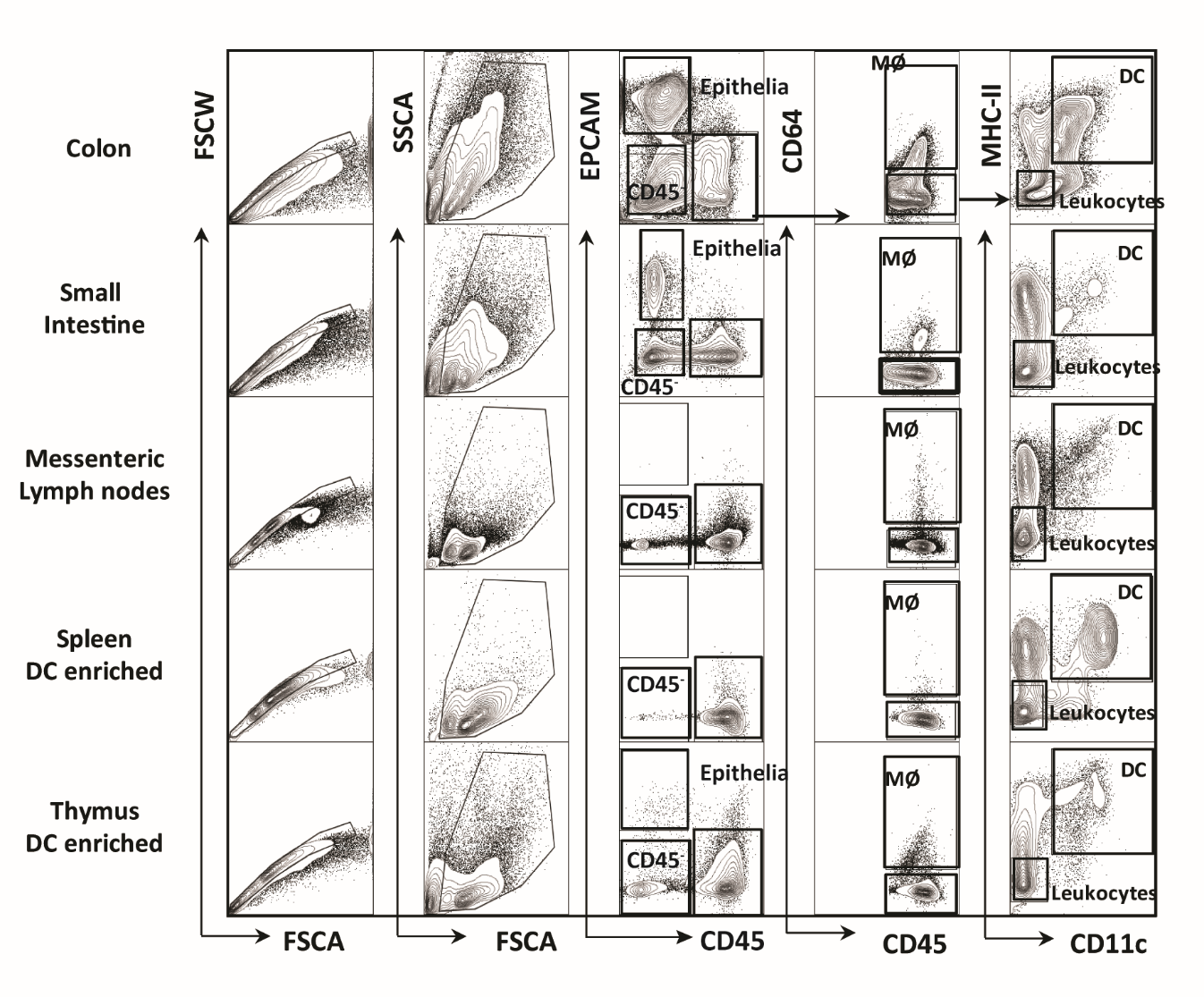


**Supplementary Material 8**: **FACS gating strategy to sort individual populations of cells from intestinal (SI and colon) and systemic (spleen, MLN and thymus) tissues.** Representative flow cytometry density plots of the sorting panels for colon, small intestine (SI), mesenteric lymph nodes (MLNs) and spleen and thymus tissues for cells sorted from WT and MyD88^-/-^ mice showing the overall gating strategy. Black squares show sorting gates. Spleen cells were enriched for dendritic cells (DCs) using mouse pan dendritic cell isolation kit (Miltenyi Biotec). Cells from thymus tissue were enriched for dendritic and epithelial cells by percoll gradient centrifugation. X axis represents forward scatter parameter (FSC) or cell surface expression of markers CD45 or CD11c as specified and Y axis represents FSC or side scatter parameter (SSC) and cell surface expression of cell specific markers such as EpCAM, CD64 or MHCII as specified. Data from MyD88^-/-^ mice, ((n=6 males, age: 23-27 weeks old). MØ- Macrophages.

**Supplementary Material 9**

**
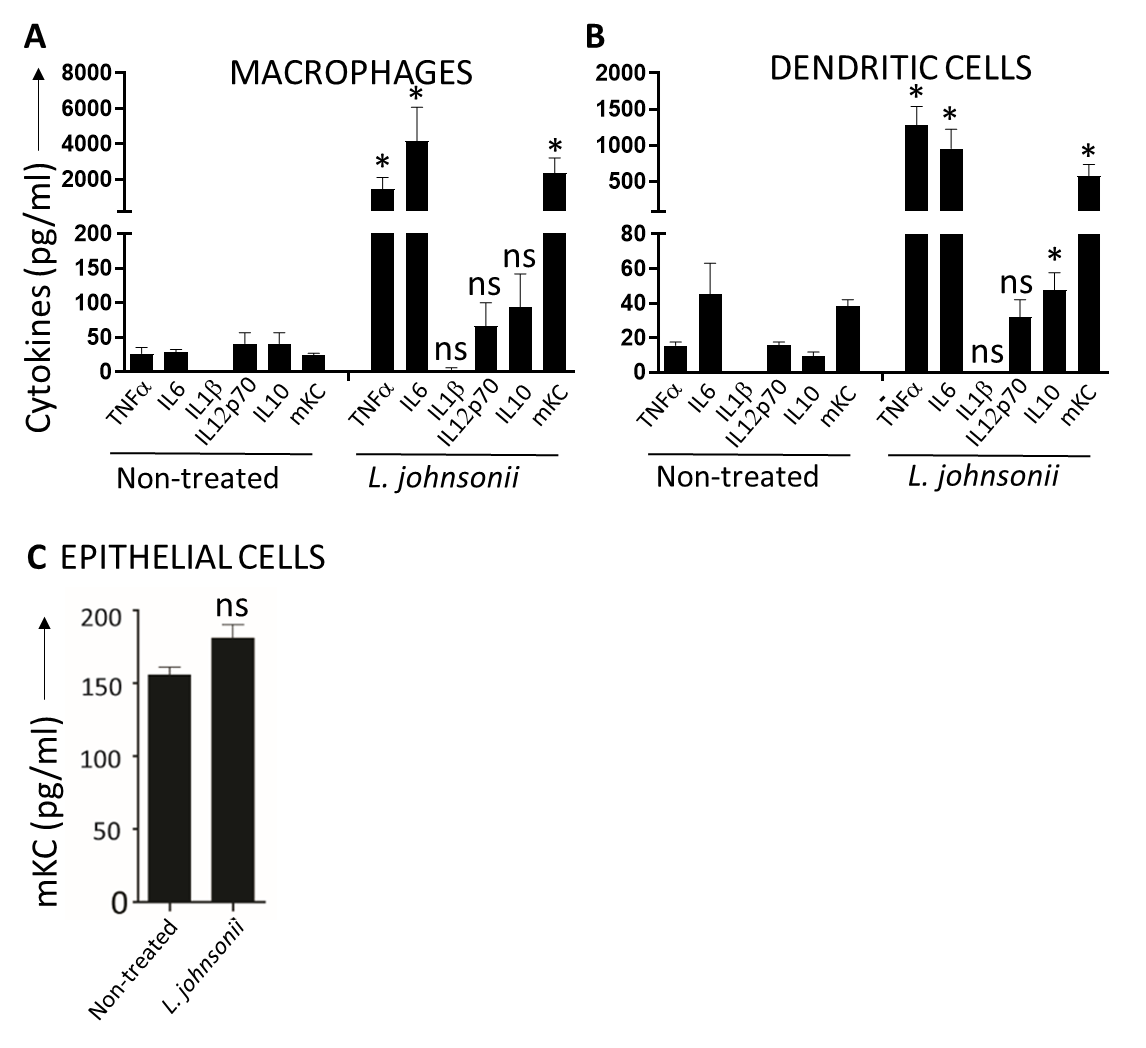
**

**Supplementary Material 9**. ***L. johnsonii* induce cytokine responses by murine macrophages and dendritic cells, but do not induce mKC response in CMT93 epithelial cells.** *L. johnsonii* was co-cultured with (A) BMDMs, (B) BMDDCs and (C) CMT93 Epithelial cells at MOI of 10 for 24 hours and supernatants were collected to analyse cytokine and chemokine, TNF-α, Il-6, Il-1b, Il-12p70, Il-10 and mKC, secretion where compared to non-treated cells that did not receive any treatment (non-treated) as indicated . Data are from three independent experiments. Statistical analyses were performed by Two- tailed Students t test. p<0.05 (denoted by *) was considered statistically significant. ns- nonsignificant.

**Supplementary Material 10**


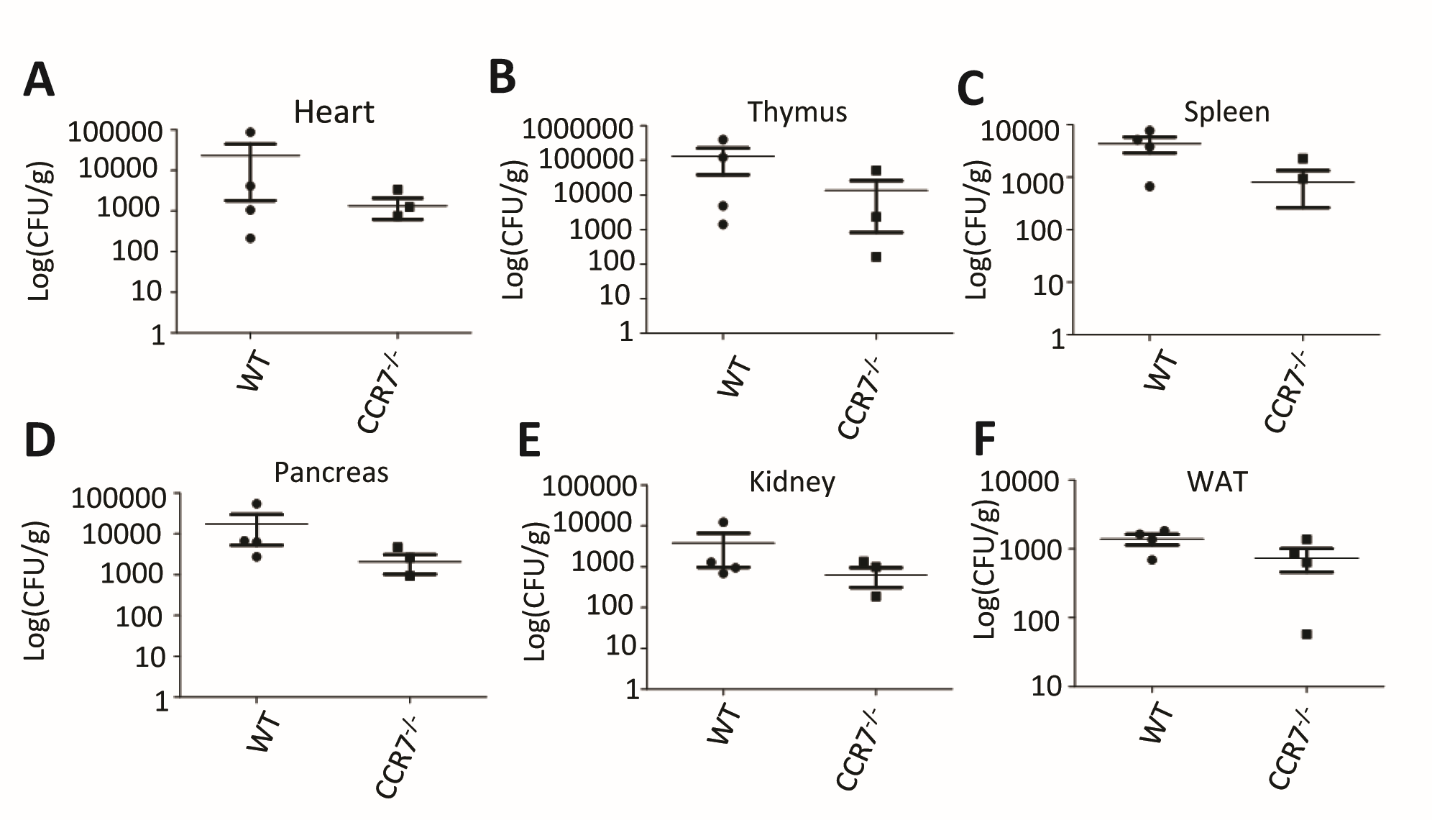


**Supplementary Material 10**. **Recovery of bacteria from systemic tissues in CCR7^-/-^ is comparable to WT mice.** (A-F) Indicated tissues were isolated from co-housed WT and CCR7^-/-^ mice, weighed in sterile tubes and screened for bacteria using workflow 2. Y axis represents CFU/g which is calculated by enumerating the total number of colonies grown in aerobic (in BHI agar) and anaerobic (in YCFA agar) plates from whole tissues homogenates of a particular tissue divided by the total weight of the tissue from each animal. No statistically significant differences were observed between Log (CFU/g) of bacterial recovery for any of the 4 WT and 4 CCR7^-/-^ mice (all males, 16 weeks old). Statistical analyses were performed by Two-tailed Students t test using GraphPad Prism.

**Supplementary Material 11**

Bacteria cultured and identified from systemic tissues of WT and CCR7^-/-^ mice.

| **Tissue** | **Strain ID** | **Genotype** | |
| --- | --- | --- | --- |
|  |  | **WT** (x/n) | **CCR7^-/-^** (x/n) |
| **Heart** | *Lactobacillus johnsonii* | 3/4 | 3/4 |
| **Thymus** | *Lactobacillus johnsonii* | 2/4 | 3/4 |
| **Liver** | *Lactobacillus johnsonii* | 3/4 | 1/4 |
|  | *Lactobacillus murinus* | 1/4 | 1/4 |
|  | *Enterococcus faecalis* | 1/4 | 1/4 |
| **Spleen** | *Lactobacillus johnsonii* | 3/4 | 2/4 |
|  | *Lactobacillus murinus* | 1/4 | 0/4 |
| **Pancreas** | *Lactobacillus johnsonii* | 3/4 | 2/4 |
|  | *Enterococcus faecalis* | 1/4 | 1/4 |
| **Kidney** | *Lactobacillus johnsonii* | 4/4 | 2/4 |
|  | *Enterococcus faecalis* | 1/4 | 1/4 |
| **MLN** | *Lactobacillus johnsonii* | 4/4 | 1/4 |
|  | *Enterococcus faecalis* | 0/4 | 1/4 |
| **WAT** | *Lactobacillus johnsonii* | 4/4 | 2/4 |
| x- No. of mice positive for bacteria, n- Total no. of mice tested | | | |

**Supplementary Material 12**

Antibodies used in immunostaining and sorting of single cell suspensions

| **Target** | **Fluorophore** | **Company** |
| --- | --- | --- |
| CD45 | APC | Biolegend |
| EPCAM | PE | Biolegend |
| CD11c | PE-Cy7 | Biolegend |
| CD64 | PE-Dazzle | Biolegend |
| FITC | MHC-II | Biolegend |
